# Association between vitamin D and inflammatory markers in the Chinese healthy adult population: a cross-sectional study

**DOI:** 10.29219/fnr.v70.14141

**Published:** 2026-07-31

**Authors:** Ye Hu, Hongxiao Li, Yi Chen, Yubei Yang, Yubo Xing, Jie Xiang, Lina Shao

**Affiliations:** 1 Geriatric Medicine Center, Department of Endocrinology, Zhejiang Provincial People’s Hospital (Affiliated People’s Hospital, Hangzhou Medical College), Hangzhou, China; 2 School of Basic Medical Sciences and Forensic Medicine, Hangzhou Medical College, Hangzhou, China; 3 Internal Medicine Department, Pujiang Hospital, Jinhua, China; 4 Urology & Nephrology Center, Department of Urology, Zhejiang Provincial People’s Hospital (Affiliated People’s Hospital, Hangzhou Medical College), Hangzhou, China; 5 Zhejiang Provincial Center for Disease Control and Prevention, Hangzhou, China; 6 Urology & Nephrology Center, Department of Nephrology, Zhejiang Provincial People’s Hospital (Affiliated People’s Hospital, Hangzhou Medical College), Hangzhou, China; 7 The Fourth School of Clinical Medicine, Zhejiang Chinese Medical University, Hangzhou, China

**Keywords:** vitamin D, systemic immune-inflammation index, platelet to lymphocyte ratio, aggregate index of systemic inflammation, inflammation

## Abstract

**Background/Objectives:**

Vitamin D (VitD) plays an essential role in immune regulation, and growing evidence associates low VitD status with elevated systemic inflammation. However, large-scale, population-based evidence linking VitD status to systemic inflammatory markers in Chinese population are lacking.

**Objective:**

This study aimed to investigate the relationship between VitD levels and inflammatory markers in healthy Chinese individuals.

**Methods:**

This cross-sectional study was conducted at Zhejiang Provincial People’s Hospital (March 2019–August 2023) including a total of 51,521 participants. VitD levels were categorized according to serum 25-hydroxyvitamin D concentrations, and inflammatory markers were derived using established formulas. Primary outcome measures included the systemic immune-inflammation index (SII), platelet-to-lymphocyte ratio (PLR), and aggregate index of systemic inflammation (AISI). Associations between VitD levels and inflammatory markers were assessed using Spearman’s correlation and linear regression.

**Results:**

SII, PLR, and AISI were significantly higher among participants with VitD deficiency (*P* < 0.05). VitD levels were negatively correlated with SII (*r* = –0.063, *P* < 0.001), PLR (*r* = –0.095, *P* < 0.001), and AISI (*r* = –0.044, *P* < 0.001). In multivariable linear regression adjusting for age, sex, low-density lipoprotein cholesterol, triglycerides, uric acid, glucose, albumin, alanine transaminase, alkaline phosphatase, serum creatinine, and hemoglobin, VitD deficiency remained independently associated with SII, PLR, and AISI.

**Conclusion:**

VitD deficiency is an independent risk factor for elevated inflammatory markers, supporting a potential immunomodulatory role of VitD and highlighting the need for further research on its potential benefits in chronic disease prevention.

## Popular scientific summary

- In this large study of over 51,000 Chinese adults, vitamin D (VitD) deficiency was common and linked to higher levels of blood markers reflecting inflammation.
- Lower VitD levels were consistently associated with greater systemic inflammatory burden.
- Because chronic low-grade inflammation contributes to many long-term diseases, maintaining adequate VitD levels may be important for overall health.
- Further studies are needed to determine whether VitD supplementation can reduce inflammation and disease risk.

Vitamin D (VitD) is a fat-soluble vitamin essential for calcium and phosphorus metabolism and bone health and for regulating various biological processes in non-skeletal systems (1). The main active form of VitD is 25-hydroxyvitamin D (25[OH]D), which exerts biological effects through VitD receptors (VDR) distributed across different tissues (2). Clinically, VitD deficiency has classically been linked to severe bone deformities, such as rickets, underscoring its importance in bone mineralization and calcium homeostasis (3). More recently, VitD has been implicated in immune modulation, with observational and mechanistic studies suggesting associations with susceptibility to autoimmune diseases and infections (4, 5). Furthermore, VitD deficiency has been linked to several chronic diseases, such as cardiovascular diseases, diabetes, and autoimmune disorders (6–8). Collectively, these findings underscore the multifaceted role of VitD in health and disease, advocating its sufficient intake to prevent associated chronic conditions (9, 10).

Converging evidence indicates that chronic, low-grade inflammation contributes to the pathogenesis of numerous diseases, particularly age-related conditions such as metabolic disorders, osteoporosis, and cardiovascular diseases (11, 12). The systemic immune-inflammation index (SII), platelet-to-lymphocyte ratio (PLR), and aggregate index of systemic inflammation (AISI) are emerging inflammatory markers calculated based on peripheral blood platelet, neutrophil, monocyte, and lymphocyte counts, comprehensively reflecting the inflammatory and immune status of the body(11, 13). Research indicates that SII assesses the immune-inflammatory state, AISI evaluates the overall inflammatory state, and PLR reflects platelet aggregation and systemic inflammation (14–17). These indicators play predictive roles in many diseases.

Recent evidence suggests that VitD contributes to the regulation of chronic low-grade inflammation (4, 18). VitD deficiency has been associated with enhanced inflammatory responses, including elevated levels of inflammatory markers across various conditions, indicating a potential role in modulating immune responses (19, 20). In addition, VitD supplementation can influence inflammatory pathways and may reduce the risk of chronic diseases associated with inflammation (21). Moreover, the influence of VitD on inflammation could provide insights into its broader implications for health, particularly in conditions such as cardiovascular diseases and metabolic syndrome (10, 22, 23).

However, large-scale evidence in Chinese population linking VitD levels to inflammatory indicators remain limited. Accordingly, we conducted this cross-sectional study to evaluate the association between VitD and inflammatory markers, including SII, PLR, and AISI, in healthy individuals. Understanding these interactions may help to inform therapeutic strategies to mitigate inflammation-related health issues. This study aimed to provide a scientific basis for recommending VitD supplementation as a strategy for mitigating inflammation-related health issues.

## Materials and methods

### Study design and participants

**Fig. 1:**
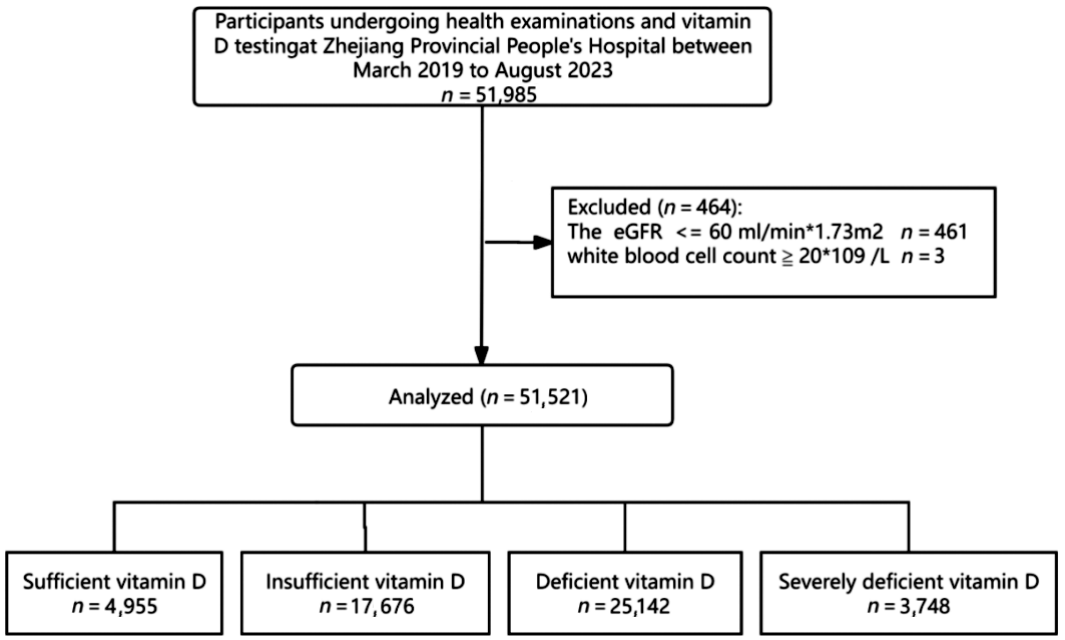
Flow chart of study: the inclusion criteria were age 18–89 years and complete VitD and blood test results. The exclusion criteria comprised eGFR < 60 mL/min/1.73 m^2^ (*n* = 461) and white blood cell count ≥ 20 × 10^9^/L (*n* = 3). Finally 51,521 participants were included. eGFR: estimated glomerular filtration rate; VitD: Vitamin D.

This cross-sectional study included individuals who underwent routine health examinations at Zhejiang Provincial People’s Hospital between March 2019 and August 2023. When individuals exhibit symptoms such as fever, cough, abdominal pain, etc., the health examination center will defer the examination and suggest seeking medical consultation at a clinic. Therefore, our research focused on the healthy individuals who underwent physical examinations. The inclusion criteria were age 18–89 years and complete VitD and blood test results. The exclusion criteria comprised estimated glomerular filtration rate (eGFR) < 60 mL/min/1.73 m^2^ (*n* = 461) and white blood cell count ≥ 20 × 10^9^ /L (*n* = 3). The screening process is shown in Fig. 1.

### Ethical approval

This study was conducted in accordance with the principles of the Declaration of Helsinki and was approved by the Ethics Committee of Zhejiang Provincial People’s Hospital (approval no.: QT2025268). The requirement for informed consent was waived owing to the retrospective nature of the study.

### Data collection

Baseline data were extracted from the hospital’s medical records system, including age, sex, and blood test results. Blood samples were collected after at least 8 h of fasting. Laboratory measurements included white blood cell count, hemoglobin, platelet count (PLT), monocyte count (MONO), neutrophil count (NEU), lymphocyte count (LYM), lipid profile (triglycerides [TG], low-density lipoprotein cholesterol [LDL-C]), liver and renal function markers (alanine transaminase [ALT], alkaline phosphatase [ALP], albumin [ALB], serum creatinine [Scr]), uric acid (UA), fasting blood glucose (FBG), and VitD levels.

### VitD group classification

VitD levels were assessed by measuring serum 25(OH)D. We defined VitD levels as follows: normal, >30 ng/mL; insufficient, 20–30 ng/mL (excluding 20 ng/mL); deficient, 10–20 ng/mL (excluding 10 ng/mL); and severely deficient, <10 ng/mL.

### Calculation of the inflammatory markers

The SII was calculated using the formula: SII = PLT × NEU / LYM; PLR as PLR = PLT / LYM; and AISI as AISI = NEU × PLT × MONO / LYM.

### Statistical analysis

All statistical analyses were conducted using IBM SPSS Statistics (Version 26.0, IBM Corp., Armonk, NY, USA). The Kolmogorov-Smirnov test was used to assess the normality of the variables. Quantitative data are presented as either means with standard deviations or medians with interquartile ranges, and qualitative data are presented as percentages. The *t*-test was used to compare data with a normal distribution, and the rank-sum test was used to assess data that did not conform to a normal distribution. The chi-square test was used to compare qualitative data. Since four groups were included in this study, Kruskal-Wallis H test (for non-normally distributed data) should be adopted for overall between-group comparisons. Associations between VitD levels and inflammatory markers was assessed using Spearman’s correlation analysis. Linear regression models were constructed to identify factors associated with inflammatory markers. Three models were constructed. Model 1 was the crude model; Model 2 was adjusted for age and sex; and Model 3 was further adjusted for LDL-C, TG, UA, FBG, ALB, ALT, ALP, Scr, and hemoglobin. All statistical tests were two-tailed, and significance was set at *P* < 0.05.

## Results

### Comparison of clinical characteristics among the four groups according to VitD levels

**Fig. 2:**
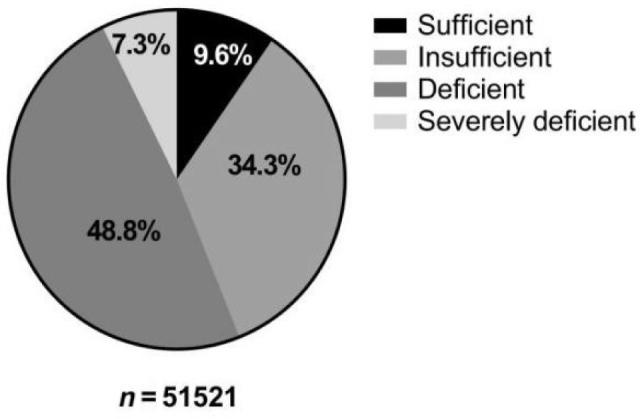
The distribution of vitamin D status among the examination population: the normal group comprised 4,955 individuals (9.6%); the insufficiency group, 17,676 (34.3%); the deficiency group, 25,142 (48.8%); and the severe deficiency group, 3,748 (7.3%).

The 51,521 patients were divided into four groups according to their 25(OH)D levels: the normal group comprised 4,955 individuals (9.6%); the insufficiency group, 17,676 (34.3%); the deficiency group, 25,142 (48.8%); and the severe deficiency group, 3,748 (7.3%). More than half of the population was VitD deficient (Fig. 2).

The clinical characteristics of the four groups are presented in Table 1. Significant differences were observed in age, sex, FBG, TG, LDL-C, UA, ALT, ALP, ALB, Scr, and inflammatory markers among the groups across VitD categories (*P* < 0.001). Inflammatory indices were higher in the severe deficiency group than in the normal group: SII [403.94 (300.65, 540.01) vs. 360.65 (267.42, 483.76)], PLR [121.26 (98.87, 150.91) vs. 108.81 (88.87, 134.70)], and AISI [138.13 (92.10, 209.16) vs. 121.75 (80.70, 184.36)]; all differences were statistically significant (*P* < 0.001).

**Table 1:** Comparison of the clinical and laboratory data between the four groups

Variables	Total (*n* = 51,521)	Sufficient group (*n* = 4,955)	Insufficient group (*n* = 17,676)	Deficient group (*n* = 25,142)	Severely deficient group (*n* = 3,748)	*P*
Age (years)	43.00 (33.00, 55.00)	54.00 (41.00, 63.00)	48.00 (37.00, 58.00)	40.00 (32.00, 51.00)	33.00 (28.00, 42.00)	< 0.001
Sex, *n* (%)						< 0.001
Female	27,611 (53.59)	3,400 (68.62)	11,351 (64.22)	11,905 (47.35)	955 (25.48)	
Male	23,910 (46.41)	1,555 (31.38)	6,325 (35.78)	13,237 (52.65)	2,793 (74.52)	
LDL-C, mmol/L	2.86 (2.37, 3.39)	2.89 (2.39, 3.43)	2.92 (2.43, 3.46)	2.84 (2.35, 3.37)	2.66 (2.18, 3.18)	< 0.001
TG, mmol/L	1.18 (0.83, 1.77)	1.28 (0.90, 1.91)	1.31 (0.91, 1.93)	1.12 (0.79, 1.69)	0.93 (0.69, 1.36)	< 0.001
UA, μmol/L	336.00 (276.00, 401.00)	353.00 (295.00, 417.00)	352.00 (294.00, 414.00)	326.00 (269.00, 394.00)	292.00 (248.00, 352.00)	< 0.001
FBG, mmol/L	4.78 (4.49, 5.14)	4.90 (4.57, 5.30)	4.84 (4.54, 5.24)	4.74 (4.46, 5.08)	4.66 (4.39, 4.95)	< 0.001
ALB, g/L	43.90 (42.30, 45.50)	43.70 (42.05, 45.40)	43.90 (42.30, 45.60)	43.90 (42.30, 45.50)	43.70 (42.10, 45.40)	< 0.001
ALT, U/L	19.00 (13.00, 29.00)	20.00 (15.00, 29.00)	20.00 (15.00, 31.00)	18.00 (12.00, 28.00)	14.00 (10.00, 22.00)	< 0.001
ALP, U/L	70.00 (58.00, 85.00)	75.00 (63.00, 89.00)	74.00 (61.00, 88.00)	68.00 (56.00, 83.00)	62.00 (52.00, 76.00)	< 0.001
Scr, μmol/L	74.00 (63.70, 84.50)	79.90 (70.10, 89.00)	77.70 (67.10, 86.60)	71.50 (62.10, 82.80)	64.80 (58.30, 74.10)	< 0.001
eGFR, ml/min/1.73 m^2^	100.11 (89.96, 110.96)	92.35 (82.88, 102.16)	96.77 (87.36, 106.35)	102.87 (92.66, 113.52)	110.73 (100.09, 120.75)	< 0.001
Hemoglobin, g/L	145.00 (133.00, 157.00)	149.00 (138.00, 159.00)	149.00 (137.00, 159.00)	143.00 (132.00, 156.00)	135.00 (127.00, 146.00)	< 0.001
SII	383.50 (287.94, 513.58)	360.65 (267.42, 483.76)	372.99 (280.25, 497.98)	394.18 (296.01, 525.90)	403.94 (300.65, 540.01)	< 0.001
PLR	114.15 (92.67, 140.58)	108.81 (88.87, 134.70)	111.02 (90.37, 136.16)	116.36 (94.40, 143.27)	121.26 (98.87, 150.91)	< 0.001
AISI	131.85 (88.36, 197.31)	121.75 (80.70, 184.36)	127.93 (85.77, 191.91)	135.90 (91.59, 201.76)	138.13 (92.10, 209.16)	< 0.001

The Kolmogorov-Smirnov test was used to assess the normality of the variables, all data did not conform to a normal distribution. Quantitative data are presented as medians with interquartile ranges, and qualitative data are presented as percentages. The rank-sum test was used to assess data that did not conform to a normal distribution. The chi-square test was used to compare qualitative data. Since four groups were included in the present study, Kruskal-Wallis H test (for non-normally distributed data) should be adopted for overall between-group comparisons. LDL-C: low-density lipoprotein cholesterol; TG: triglycerides; UA: uric acid; FBG: fasting blood glucose; ALB: albumin; ALT: alanine transaminase; ALP: alkaline phosphatase; Scr: serum creatinine; eGFR: estimated glomerular filtration rate, calculated using the 2009 Chronic Kidney Disease Epidemiology Collaboration (CKD-EPI) formula; SII: systemic immune-inflammation index; PLR: platelet to lymphocyte ratio; AISI: aggregate index of systemic inflammation

### Correlations between VitD and inflammatory markers

**Fig. 3:**
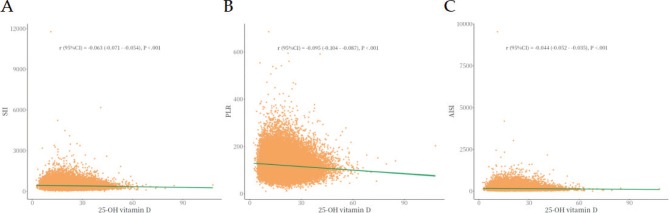
Correlations between VitD and Inflammatory Markers using Spearman’s correlation analysis. (A) Correlation between VitD and SII: a significant negatively correlation between 25-OH VitD levels and SII (*r* = -0.063, *P* < 0.001); (B) Correlation between VitD and PLR: a significant negatively correlation between 25-OH VitD levels and PLR (*r* = -0.095, *P* < 0.001); (C) Correlation between VitD and AISI: a significant negatively correlation between 25-OH VitD levels and AISI (*r* = -0.044, *P* < 0.001). VitD: Vitamin D; 25-OH VitD: 25-hydroxyvitamin D; SII: systemic immune-inflammation index; PLR: platelet to lymphocyte ratio; AISI: aggregate index of systemic inflammation.

To further assess the relationship between VitD levels and inflammatory markers, we performed Spearman’s correlation analysis. The results showed that 25(OH)D levels were significant negatively correlated with SII (*r* = –0.063, *P* < 0.001), PLR (*r* = –0.095, *P* < 0.001), and AISI (*r* = –0.044, *P* < 0.001) (Fig. 3).

### Association between VitD levels and SII

Linear regression analysis was performed to assess the factors associated with inflammatory markers. With SII as the dependent variable, the crude model showed that insufficient (β = 9.93, 95% CI = 3.06–16.79, *P* = 0.005), deficient (β = 32.21, 95% CI = 25.57–38.85, *P* < 0.001) and severely deficient VitD levels (β = 42.48, 95% CI = 33.23–51.73, *P* < 0.001) were associated with SII. After adjusting for age and sex, deficient and severely deficient VitD levels remained associated with SII. Further adjustment for LDL-C, TG, UA, FBG, ALB, ALT, ALP, Scr, and hemoglobin showed that deficient (β = 16.64, 95% CI = 9.80–23.48, *P* < 0.001) and severely deficient VitD levels (β = 15.33, 95% CI = 5.67–25.00, *P* = 0.002) remained independently associated with SII (Table 2).

**Table 2:** Linear regression analysis assessing the association between VitD levels and SII

Variables	Model 1		Model 2		Model 3	
	β (95% CI)	*P*	β (95% CI)	*P*	β (95% CI)	*P*
Sufficient	0.00 (Reference)		0.00 (Reference)		0.00 (Reference)	
Insufficient	9.93 (3.06 ~ 16.79)	0.005	5.94 (–0.95 ~ 12.82)	0.091	3.60 (–3.26 ~ 10.45)	0.304
Deficient	32.21 (25.57 ~ 38.85)	< 0.001	19.89 (13.04 ~ 26.74)	< 0.001	16.64 (9.80 ~ 23.48)	< 0.001
Severely deficient	42.48 (33.23 ~ 51.73)	< 0.001	20.84 (11.18 ~ 30.51)	< 0.001	15.33 (5.67 ~ 25.00)	0.002

Model1: Crude.

Model2: Adjust for age and sex.

Model3: Model2 + Adjust for LDL-C, TG, UA, FBG, ALB, ALT, ALP, Scr, and hemoglobin.

LDL-C: low-density lipoprotein cholesterol; TG: triglycerides; UA: uric acid; FBG: fasting blood glucose; ALB: albumin; ALT: alanine transaminase; ALP: alkaline phosphatase; Scr: serum creatinine; SII: systemic immune-inflammation index; VitD: Vitamin D.

### Association between VitD levels and AISI

With AISI as the dependent variable, the crude model showed that insufficient (β = 4.05, 95% CI = 0.16–7.94, *P* = 0.041), deficient (β = 12.68, 95% CI = 8.92–16.44, *P* < 0.001) and severely deficient VitD levels (β = 16.39, 95% CI = 11.16–21.63, *P* < 0.001) were associated with AISI. After adjusting for age and sex, VitD deficiency and severe deficiency remained associated with AISI. Further adjustment for LDL-C, TG, UA, FBG, ALB, ALT, ALP, Scr, and hemoglobin showed that deficient (β = 11.76, 95% CI = 7.91–15.61, *P* < 0.001) and severely deficient VitD levels (β = 18.31, 95% CI = 12.87–23.76, *P* < 0.001) remained independently associated with AISI (Table 3).

**Table 3:** Linear regression analysis assessing the association between VitD levels and AISI

Variables	Model 1		Model 2		Model 3	
	β (95% CI)	*P*	β (95% CI)	*P*	β (95% CI)	*P*
Sufficient	0.00 (Reference)		0.00 (Reference)		0.00 (Reference)	
Insufficient	4.05 (0.16 ~ 7.94)	0.041	3.57 (–0.32 ~ 7.46)	0.072	1.74 (–2.12 ~ 5.60)	0.378
Deficient	12.68 (8.92 ~ 16.44)	< 0.001	13.67 (9.80 ~ 17.54)	< 0.001	11.76 (7.91 ~ 15.61)	< 0.001
Severely deficient	16.39 (11.16 ~ 21.63)	< 0.001	19.99 (14.52 ~ 25.45)	< 0.001	18.31 (12.87 ~ 23.76)	< 0.001

Model1: Crude.

Model2: Adjust for age and sex.

Model3: Model2 + Adjust for LDL-C, TG, UA, FBG, ALB, ALT, ALP, Scr, and hemoglobin.

LDL-C: low-density lipoprotein cholesterol; TG: triglycerides; UA: uric acid; FBG: fasting blood glucose; ALB: albumin; ALT: alanine transaminase; ALP: alkaline phosphatase; Scr: serum creatinine; AISI: aggregate index of systemic inflammation; VitD: Vitamin D.

### Association between VitD levels and PLR

With PLR as the dependent variable, the crude model showed that insufficient (β = 1.77, 95% CI = 0.51–3.03, *P* = 0.006), deficient (β = 7.38, 95% CI = 6.16–8.61, *P* < 0.001) and severely deficient VitD levels (β = 13.89, 95% CI = 12.19–15.59, *P* < 0.001) were associated with PLR. After adjusting for age and sex, deficient and severely deficient VitD levels remained associated with PLR. Further adjustment for LDL-C, TG, UA, FBG, ALB, ALT, ALP, Scr, and hemoglobin showed that deficient (β = 1.91, 95% CI = 0.70–3.13, *P* < 0.001) and severely deficient VitD levels (β = 2.27, 95% CI = 0.56–3.98, *P* < 0.001) remained independently associated with PLR (Table 4).

**Table 4:** Linear regression analysis assessing the association between VitD levels and PLR

Variables	Model 1		Model 2		Model 3	
	β (95% CI)	*P*	β (95% CI)	*P*	β (95% CI)	*P*
Sufficient	0.00 (Reference)		0.00 (Reference)		0.00 (Reference)	
Insufficient	1.77 (0.51 ~ 3.03)	0.006	0.67 (–0.57 ~ 1.92)	0.288	0.60 (–0.61 ~ 1.81)	0.333
Deficient	7.38 (6.16 ~ 8.61)	< 0.001	2.98 (1.74 ~ 4.22)	< 0.001	1.91 (0.70 ~ 3.13)	0.002
Severely deficient	13.89 (12.19 ~ 15.59)	< 0.001	5.39 (3.64 ~ 7.13)	< 0.001	2.27 (0.56 ~ 3.98)	0.009

Model1: Crude.

Model2: Adjust for age and sex.

Model3: Model2 + Adjust for LDL-C, TG, UA, FBG, ALB, ALT, ALP, Scr, and hemoglobin.

LDL-C: low-density lipoprotein cholesterol; TG: triglycerides; UA: uric acid; FBG: fasting blood glucose; ALB: albumin; ALT: alanine transaminase; ALP: alkaline phosphatase; Scr: serum creatinine; PLR: platelet to lymphocyte ratio; VitD: Vitamin D.

## Discussion

VitD deficiency is highly prevalent worldwide (24). In this study, only 9.62% of participants had sufficient VitD levels, consistent with prior surveys. Wan et al. reported that among 6,329 patients with type 2 diabetes, 81.9% had insufficient VitD levels and 46.6% had VitD deficiency (25). Similarly, Hu et al. found that 71.74% of 22,130 individuals in the US population had insufficient VitD levels (26). A growing body of evidence links low VitD status to chronic disease risk and to alterations in immune and inflammatory pathways. However, few studies have investigated the relationship between VitD and systemic inflammatory markers.

Patients with VitD deficiency often exhibit higher levels of inflammatory markers, suggesting a more pronounced inflammatory state and a potential link between VitD deficiency and heightened inflammatory response (27, 28). Okuyan et al. demonstrated that the SII was significantly increased in children with VitD deficiency. Collectively, these findings support an association between VitD deficiency and elevated systemic inflammatory markers, consistent with the proposed immunomodulatory and anti-inflammatory properties of VitD (20). Ganji et al. found elevated C-reactive protein (CRP) levels in American adults with VitD deficiency (29). Additionally, Bueloni et al. conducted a randomized, double-blind, placebo-controlled trial, which revealed that VitD supplementation improved immune-inflammatory biomarkers in young postmenopausal women (30). These studies indicate that individuals with severe VitD deficiency typically exhibit higher levels of inflammation. However, most previous studies have been conducted in Western populations or specific groups. To our knowledge, this study is the first to explore the relationship between VitD status and three systemic inflammatory markers in a large sample of apparently healthy Chinese adults.

We conducted a cross-sectional analysis of 51,521 healthy individuals undergoing physical examinations, representing the first study to systematically evaluate the relationship between VitD levels and systemic inflammatory markers (SII, PLR, and AISI). The results showed that the levels of all three inflammatory indicators in individuals with severe VitD deficiency were significantly higher than in those with normal VitD levels, consistent with findings of previous studies. Although the Spearman’s correlation coefficient between VitD and inflammatory indicators is relatively weak, given that the subjects in this study were all healthy individuals who underwent routine physical examinations, their systemic inflammatory markers were typically within the normal range. These numerical ranges are relatively narrow, which may limit the significance of the correlation coefficient. Moreover, systemic inflammation is a complex characteristic influenced by multiple factors, and the VitD level may only account for a small part of the variability. However, it still suggests a significant contribution. More importantly, the linear regression analyses further confirmed that, even after adjusting for multiple potential confounding factors, severely deficient VitD levels remained independently associated with elevated SII (β = 15.33), PLR (β = 2.27), and AISI (β = 18.31).

These findings have several significant clinical implications. Firstly, they suggest that VitD deficiency may be a modifiable factor for chronic low-grade inflammation. VitD is widely expressed in immune cells through VDR, which can inhibit the production of pro-inflammatory cytokines (such as tumor necrosis factor-α (TNF-α) and interleukin-6 (IL-6)) and promote the release of anti-inflammatory factors such as IL-10 (30, 31). Therefore, VitD deficiency may lead to an imbalance in immune regulation, thereby promoting systemic inflammatory responses (2, 4). The elevated SII, PLR, and AISI observed in this study are manifestations of this inflammatory state in the blood cell components. Secondly, this study provides insights into the potential role of VitD in the prevention and treatment of chronic diseases. VitD deficiency is closely associated with various inflammation-related diseases, such as cardiovascular diseases, type 2 diabetes, metabolic dysfunction-associated fatty liver disease, and autoimmune diseases (29, 32–34). By influencing systemic inflammatory pathways, VitD may affect the onset and progression of these diseases. Therefore, timely VitD supplementation in individuals with VitD deficiency may help to improve bone health and reduce the risk of related chronic diseases by attenuating chronic inflammation. Although VitD is not the main driver of inflammation in healthy individuals, its independent effect is measurable and may be of great significance for the prevention of long-term chronic diseases.

Our findings suggest that severe VitD deficiency is independently associated with SII, AISI and PLR, the strength of these association differs: the effect was strongest on AISI (β = 18.31), followed by SII (β = 15.33), and weakest on PLR (β = 2.27). We speculate that these differences may reflect distinct pathways and cellular targets underlying the anti-inflammatory effects of VitD. AISI showed the strongest correlation, likely because its formula incorporates monocytes. Monocytes, or macrophages, are the major producers of core pro-inflammatory factors such as TNF-α and IL-6 and express high levels of VDR, making them the primary targets of VitD action. The strong correlation between VitD deficiency and higher AISI may therefore indicate that the innate immune inflammatory pathway mediated by monocytes are particularly sensitive to VitD status. The association between VitD deficiency and SII suggests that VitD not only inhibits monocytes but also suppresses neutrophil chemotaxis and neutrophil extracellular traps (NET) formation and regulates the balance of lymphocyte subsets. The strength of the association between VitD deficiency and PLR significantly weakened after adjusting for factors such as age and metabolic indicators, suggesting that the impact of VitD on this pathway may largely be achieved indirectly by improving the overall metabolic environment. Its direct independent effect is weaker than its influence on AISI.

However, this study had several limitations. Firstly, because of the cross-sectional study design, we could not establish a causal relationship between VitD and inflammatory markers. Secondly, although the sample size was large, participants were recruited from a single medical center, which may introduced selection bias and limit generalizability. In addition, data on key determinants of VitD status, such as sunlight exposure and VitD supplementation, were unavailable. Future studies should employ prospective cohorts or randomized controlled trials to determine whether VitD supplementation can directly reduce the levels of these inflammatory markers and to establish the ideal blood drug concentration for anti-inflammatory effects. In addition, incorporating more detailed immunological indicators would further clarify the target points of VitD in immune-mediated inflammation.

## Conclusions

VitD levels were independently associated with SII, AISI, and PLR in this Chinese population. These findings underscore the potential role of VitD in regulating inflammatory responses, particularly in chronic diseases caused by immune dysregulation. Future studies should focus on multicenter, randomized controlled trials with larger cohorts to validate these findings and explore the long-term effects of VitD supplementation on chronic disease prevention. Further elucidation of the mechanisms underlying the immunomodulatory effects of VitD is essential for developing targeted therapeutic strategies.

## Data Availability

The original contributions presented in this study are included in the article. Further inquiries can be directed to the corresponding authors.

## References

[1] Wintermeyer E, Ihle C, Ehnert S, Stöckle U, Ochs G, Zwart P, et al. Crucial role of vitamin D in the musculoskeletal system. Nutrients 2016; 8(6):319. doi: 10.3390/nu8060319 27258303 PMC4924160

[2] Demay MB, Pittas AG, Bikle DD, Diab DL, Kiely ME, Lazaretti-Castro M, et al. Vitamin D for the prevention of disease: an endocrine society clinical practice guideline. J Clin Endocrinol Metab 2024; 109(8): 1907–47. doi: 10.1210/clinem/dgae290 38828931

[3] Holick MF. Vitamin D and bone health: what vitamin D can and cannot do. Adv Food Nutr Res 2024; 109: 43–66. doi: 10.1016/bs.afnr.2024.04.002 38777417

[4] Hewison M. COVID-19 and our understanding of vitamin D and immune function. J Steroid Biochem Mol Biol 2025; 249: 106710. doi: 10.1016/j.jsbmb.2025.106710 39986580

[5] Eder K, Grundmann SM. Vitamin D in dairy cows: metabolism, status and functions in the immune system. Arch Anim Nutr 2022; 76(1): 1–33. doi: 10.1080/1745039X.2021.2017747 35249422

[6] Faridi KF, Zhao D, Martin SS, Lupton JR, Jones SR, Guallar E, et al. Serum vitamin D and change in lipid levels over 5 y: the atherosclerosis risk in communities study. Nutrition 2017; 38: 85–93. doi: 10.1016/j.nut.2017.01.008 28526388 PMC5443111

[7] Wimalawansa SJ. Vitamin D and cardiovascular diseases: causality. J Steroid Biochem Mol Biol 2018; 175: 29–43. doi: 10.1016/j.jsbmb.2016.12.016 28027913

[8] Liu Y, Wang W, Yang Y, Deng J, Zhang Z. Vitamin D and bone health: from physiological function to disease association. Nutr Metab (Lond) 2025; 22(1): 113. doi: 10.1186/s12986-025-01011-1 41039476 PMC12490156

[9] Ellison DL, Moran HR. Vitamin D: vitamin or hormone? Nurs Clin North Am 2021; 56(1): 47–57. doi: 10.1016/j.cnur.2020.10.004 33549285

[10] Zhao S, Qian F, Wan Z, Chen X, Pan A, Liu G. Vitamin D and major chronic diseases. Trends Endocrinol Metab 2024; 35(12): 1050–61. doi: 10.1016/j.tem.2024.04.018 38824035

[11] Nøst TH, Alcala K, Urbarova I, Byrne KS, Guida F, Sandanger TM, et al. Systemic inflammation markers and cancer incidence in the UK Biobank. Eur J Epidemiol 2021; 36(8): 841–8. doi: 10.1007/s10654-021-00752-6 34036468 PMC8416852

[12] Walther K, Gröger S, Vogler JAH, Wöstmann B, Meyle J. Inflammation indices in association with periodontitis and cancer. Periodontol 2000 2024; 96(1): 281–315. doi: 10.1111/prd.12612 39317462 PMC11579835

[13] Fu C, Chen J, Wang Y, Yang Y, Li X, Liu K. Association between complete blood cell count-derived inflammatory biomarkers and gallstones prevalence in American adults under 60 years of age. Front Immunol 2024; 15: 1497068. doi: 10.3389/fimmu.2024.1497068 39867880 PMC11757271

[14] Wang R, Wen W, Jiang Z, Du Z, Ma Z, Lu A, et al. The clinical value of neutrophil-to-lymphocyte ratio (NLR), systemic immune-inflammation index (SII), platelet-to-lymphocyte ratio (PLR) and systemic inflammation response index (SIRI) for predicting the occurrence and severity of pneumonia in patients with intracerebral hemorrhage. Front Immunol 2023; 14: 1115031. doi: 10.3389/fimmu.2023.1115031 36860868 PMC9969881

[15] Altintas O, Altintas MO, Tasal A, Kucukdagli OT, Asil T. The relationship of platelet-to-lymphocyte ratio with clinical outcome and final infarct core in acute ischemic stroke patients who have undergone endovascular therapy. Neurol Res 2016; 38(9): 759–65. doi: 10.1080/01616412.2016.1215030 27477691

[16] Islam MM, Satici MO, Eroglu SE. Unraveling the clinical significance and prognostic value of the neutrophil-to-lymphocyte ratio, platelet-to-lymphocyte ratio, systemic immune-inflammation index, systemic inflammation response index, and delta neutrophil index: an extensive literature review. Turk J Emerg Med 2024; 24(1): 8–19. doi: 10.4103/tjem.tjem_198_23 38343523 PMC10852137

[17] Famakin BM. The immune response to acute focal cerebral ischemia and associated post-stroke immunodepression: a focused review. Aging Dis 2014; 5(5): 307–26. doi: 10.14336/AD.2014.0500307 25276490 PMC4173797

[18] Giustina A, Bilezikian JP, Adler RA, Banfi G, Bikle DD, Binkley NC, et al. Consensus statement on vitamin D status assessment and supplementation: whys, whens, and hows. Endocr Rev 2024; 45(5): 625–54. doi: 10.1210/endrev/bnae009 38676447 PMC11405507

[19] Chan F, Cui C, Peng Y, Liu Z. The associations among serum vitamin D concentration, systemic immune-inflammation index, and lifestyle factors in Chinese adults: a cross-sectional analysis. Front Nutr 2025; 12: 1543925. doi: 10.3389/fnut.2025.1543925 40521355 PMC12162998

[20] Okuyan O, Dumur S, Elgormus N, Uzun H. The relationship between vitamin D, inflammatory markers, and insulin resistance in children. Nutrients 2024; 16(17): 3005. doi: 10.3390/nu16173005 39275320 PMC11396811

[21] National Heart, Lung, and Blood Institute PETAL Clinical Trials Network, Ginde AA, Brower RG, et al. Early high-dose vitamin D3 for critically ill, vitamin D-deficient patients. N Engl J Med 2019; 381(26): 2529–40. doi: 10.1056/NEJMoa1911124 31826336 PMC7306117

[22] Park JE, Pichiah PBT, Cha Y. Vitamin D and metabolic diseases: Growing roles of vitamin D. J Obes Metab Syndr 2018; 27(4): 223–32. doi: 10.7570/jomes.2018.27.4.223 31089567 PMC6513299

[23] Pál É, Ungvári Z, Benyó Z, Várbíró S. Role of vitamin D deficiency in the pathogenesis of cardiovascular and cerebrovascular diseases. Nutrients 2023; 15(2): 334. doi: 10.3390/nu15020334 36678205 PMC9864832

[24] Pludowski P, Takacs I, Boyanov M, Belaya Z, Diaconu C, Mokhort T, et al. Clinical practice in the prevention, diagnosis and treatment of vitamin D deficiency: a central and Eastern European expert consensus statement. Nutrients 2022; 14(7): 1483. doi: 10.3390/nu14071483 35406098 PMC9002638

[25] Wan Z, Guo J, Pan A, Chen C, Liu L, Liu G. Association of serum 25-hydroxyvitamin D concentrations with all-cause and cause-specific mortality among individuals with diabetes. Diabetes Care 2021; 44(2): 350–7. doi: 10.2337/dc20-1485 33168652

[26] Hu Y, Gao F, Yang Y, Yang W, He H, Zhou J, et al. Serum 25(OH)D levels and mortality risk among middle-aged and elderly populations in the U.S.: a prospective cohort study. PLoS One 2025; 20(7): e0328907. doi: 10.1371/journal.pone.0328907 40705722 PMC12289007

[27] Sharif-Askari FS, Hafezi S, Sharif-Askari NS, Alsayed HAH, Mdkhana B, Selvakumar B, et al. Vitamin D modulates systemic inflammation in patients with severe COVID-19. Life Sci 2022; 307: 120909. doi: 10.1016/j.lfs.2022.120909 36028169 PMC9398944

[28] Carboo JA, Malan L, Lombard M, Nienaber A, Dolman-Macleod RC. The relationship between 25-hydroxyvitamin D and markers of intestinal and systemic inflammation in undernourished and non-undernourished children, 6–59 months. Cytokine 2025: 185: 156807. doi: 10.1016/j.cyto.2024.156807 39550924

[29] Ganji V, Tangpricha V, Zhang X. Serum vitamin D concentration ≥75 nmol/L is related to decreased cardiometabolic and inflammatory biomarkers, metabolic syndrome, and diabetes; and increased cardiorespiratory fitness in US adults. Nutrients 2020; 12(3): 730. doi: 10.3390/nu12030730 32164233 PMC7146199

[30] Bueloni-Dias FN, Orsatti CL, Cangussu LM, Poloni PF, Spadoto-Dias D, Nahas-Neto J, et al. Isolated vitamin D supplementation improves the immune-inflammatory biomarkers in younger postmenopausal women: a randomized, double-blind, placebo-controlled trial. Menopause 2018; 25(8): 897–903. doi: 10.1097/GME.0000000000001106 29738417

[31] Abdelghany AH, BaSalamah MA, Idris S, Ahmad J, Refaat B. The fibrolytic potentials of vitamin D and thymoquinone remedial therapies: insights from liver fibrosis established by CCl4 in rats. J Transl Med 2016; 14(1): 281. doi: 10.1186/s12967-016-1040-4 27681697 PMC5041560

[32] Zhao H, Tang Y, Zheng C, Ren L, Song G. Vitamin D status is independently associated with insulin resistance in patients with type 2 diabetes mellitus. Risk Manag Healthc Policy 2021; 14: 1393–9. doi: 10.2147/RMHP.S299963 33854388 PMC8039193

[33] Zittermann A, Trummer C, Theiler-Schwetz V, Lerchbaum E, März W, Pilz S. Vitamin D and cardiovascular disease: an updated narrative review. Int J Mol Sci 2021; 22(6): 2896. doi: 10.3390/ijms22062896 33809311 PMC7998446

[34] Barchetta I, Cimini FA, Cavallo MG. Vitamin D and metabolic dysfunction-associated fatty liver disease (MAFLD): an update. Nutrients 2020; 12(11): 3302. doi: 10.3390/nu12113302 33126575 PMC7693133

